# Fluid Caustics

**Published:** 1845-07

**Authors:** 


*Fluid caustics.*—M. Cazenave frequently makes use of a solution of the Sulphate of Copper for the removal of those small warts that often form upon the shoulders and back, and also of certain pediculated horny productions, which occasionally appear upon these parts.

A stronger solution must be used for the latter form of cuticular excrescence.

In the treatment of Favus and Tinea, he recommends a weak solution either of this salt of Copper, or of the nitrate of silver, or of Acetic Acid.

Of fluid caustics, one of the most potent and useful is the acid nitrate of Mercury. When used to the surface pure and undiluted, it acts as a mere caustic; but when considerably weakened, and especially when applied to a large surface, it is unquestionably absorbed, and then it acts on the system.

It usually causes a good deal of pain and inflammatory swelling. The cases most benefitted by its application are those of Lupus, in which the ulceration is extensive and not deep-seated.

The erysipelatous inflammation, which this as well as other caustics—more especially the arsenical paste—are apt to produce, need not be much dreaded; nay, the effects of the cutaneous Phlegmasia seem sometimes to be decidedly salutary in the end.—*Annalen des Maladies de la Peau.*

M. Gibert has recorded in a recent No. (Oct. 1844,) of the Revue Medicale, a case of severe scrofulous Lupus of the face, in which the progress of the disease was arrested and the extensive ulcerated surface became cicatrised under the employment, external as well as internal, of cod-liver oil. The use of this medicine was steadily persevered in for a full year and a half. During this time not only did the local malady become healed, but the general health—which had formerly been very weak and ailing—was very decidedly improved.

The patient was a young woman, and the disease had existed for nearly six years. On one occasion, she had derived very considerable benefit from the internal administration of the deuto-ioduret of Mercury, and the external use of the proto-ioduret ointment; but the benefit was temporary only. She had been subjected to a regular and protracted course of Iodine treatment; but certainly with no advantage.—*Med. Chir. Rev.*

